# Sex Differences in the Prognostic Value of Circulating Biomarkers in Patients Presenting With Acute Chest Pain

**DOI:** 10.1016/j.jacadv.2024.101567

**Published:** 2025-01-21

**Authors:** Gard Mikael Sæle Myrmel, Nasir Saeed, Ole Thomas Steiro, Hilde Lunde Tjora, Jørund Langørgen, Rune Oskar Bjørneklett, Øyvind Skadberg, Vernon Vijay Singha Bonarjee, Øistein Rønneberg Mjelva, Eva Ringdal Pedersen, Kjell Vikenes, Torbjørn Omland, Kristin Moberg Aakre

**Affiliations:** a Department of Heart Disease, Haukeland University Hospital, Bergen, Norway; b Department of Clinical Science, University of Bergen, Bergen, Norway; c Emergency Care Clinic, Haukeland University Hospital, Bergen, Norway; d Department of Clinical Medicine, University of Bergen, Bergen, Norway; e Laboratory of Medical Biochemistry, Stavanger University Hospital, Stavanger, Norway; f Department of Cardiology, Stavanger University Hospital, Stavanger, Norway; g Department of Medicine, Stavanger University Hospital, Stavanger, Norway; h K.G. Jebsen Centre for Cardiac Biomarkers, Institute of Clinical Medicine, University of Oslo, Oslo, Norway; i Department of Cardiology, Akershus University Hospital, Oslo, Norway; j Department of Medical Biochemistry and Pharmacology, Haukeland University Hospital, Bergen, Norway

**Keywords:** acute chest pain, biomarkers, risk stratification, sex differences

## Abstract

**Background:**

Biomarkers are used for long-term risk prediction of cardiovascular (CV) events in patients presenting with suspected acute coronary syndromes.

**Objectives:**

This study investigated whether there are sex differences in the long-term prognostic value of biomarkers in patients presenting with suspected non-ST-segment elevation acute coronary syndrome (NSTE-ACS).

**Methods:**

High-sensitivity cardiac troponin (hs-cTn), hs-cTnI, N-terminal pro-B-type natriuretic peptide (NT-proBNP), growth differentiation factor (GDF)-15, and C-reactive protein (CRP) concentrations were measured in 1,476 patients admitted with suspected NSTE-ACS. Patients were followed up for a median of 1,547 (IQR: 873-1,842) days until a primary composite endpoint of all-cause mortality, incident myocardial infarction, or heart failure hospitalization. A secondary endpoint of CV death was also registered.

**Results:**

For the primary endpoint, a log2 increase of hs-cTn and hs-cTnI concentration was associated with a higher adjusted hazard ratio in women (hs-cTn: 1.3, 95% CI: 1.2-1.5; hs-cTnI: 1.2, 95% CI: 1.1-1.2) than in men (hs-cTn: 1.1, 95% CI: 1.0-1.2; hs-cTnI: 1.0, 95% CI: 1.0-1.1); *P* value for interaction with sex: 0.009 (hs-cTn) and 0.005 (hs-cTnI). A similar interaction was shown for NT-proBNP (*P* for interaction: 0.043). GDF-15 and CRP were independent predictors of the primary endpoint, but the interaction by sex was nonsignificant.

**Conclusions:**

In contrast to CRP and GDF-15, increasing concentrations of hs-cTn, hs-cTnI, and NT-proBNP are associated with higher risk of death and CV events in female than in male patients presenting with suspected NSTE-ACS. Sex-adjustment of hs-cTn and NT-proBNP may increase the accuracy of long-term CV prognostication in women and men.

## Central Illustration

**Figure:**
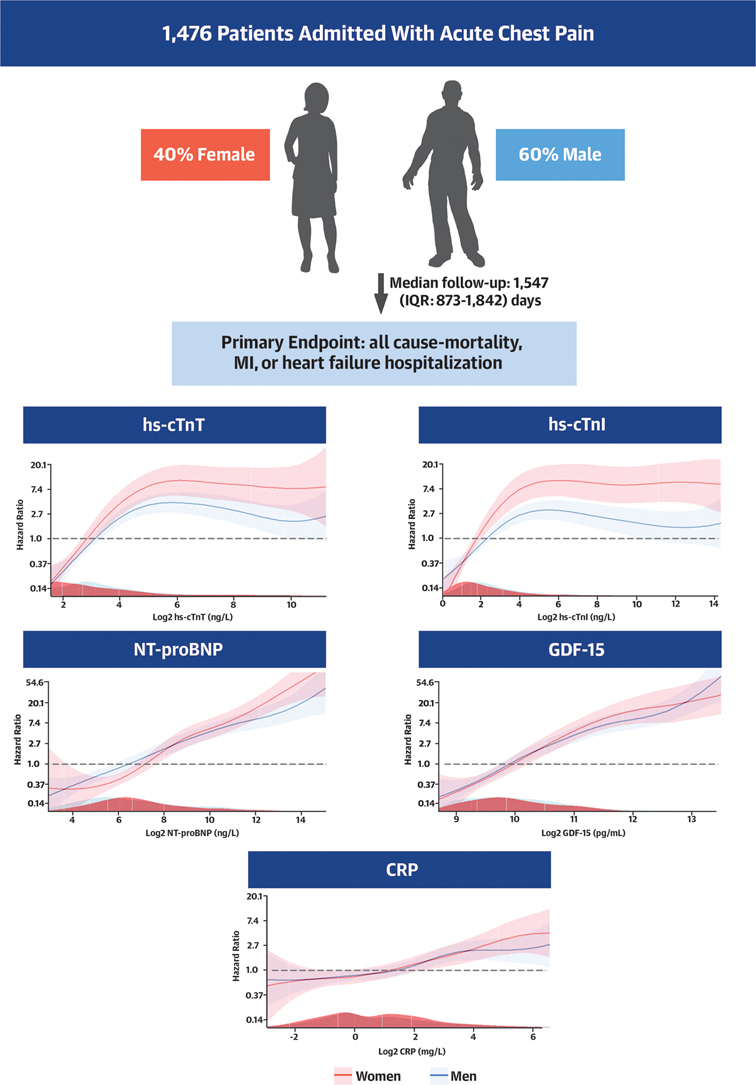

Sex differences in clinical presentation, pathophysiology, treatment, and outcomes have been reported in patients presenting with acute chest pain and suspected acute coronary syndromes (ACS).^1^, ^2^, ^3^ Conditions such as myocardial infarction (MI) with non-obstructive coronary artery disease, spontaneous coronary artery dissection, microvascular dysfunction, and takotsubo cardiomyopathy exhibit a higher prevalence among women presenting with acute chest pain compared to men.^1^ Furthermore, women also present at an older age, have more comorbidities, and tend to have a greater bleeding risk.^1^^,^^4^ Recognizing sex differences can be important when approaching risk stratification for future cardiovascular (CV) disease.

Circulating biomarkers are commonly employed for risk stratification in patients presenting with acute chest pain and suspected ACS, and cardiac injury markers such as cardiac troponins (cTn) are included in prognostic risk calculators such as the History, Electrocardiogram, Age, Risk factors, and Troponin (HEART), Global Registry of Acute Coronary Events (GRACE), and Thrombolysis In Myocardial Infarction (TIMI) scores (History, Electrocardiogram, Age, Risk factors, and Troponin), GRACE, and TIMI scores. None of these risk calculators apply sex-specific cTn cutoff values for risk prediction. Moreover, numerous studies have shown that circulating biomarkers other than cardiac injury markers can be used for predicting mortality and CV events among patients presenting with acute chest pain, including N-terminal pro-B-type natriuretic peptide (NT-proBNP), growth differentiation factor (GDF)-15, and C-reactive protein (CRP).^5^, ^6^, ^7^

Several studies have demonstrated that cTn are stronger long-term predictors of CV events in women than in men in the general population.^8^^,^^9^ However, there is limited knowledge regarding sex differences in the long-term prognostic value of cTn, NT-proBNP, GDF-15, and CRP in patients presenting with suspected non-ST-segment elevation acute coronary syndrome (NSTE-ACS). Recognizing sex differences in the prognostic value of biomarkers and using sex-specific cutoffs in risk calculators may aid in more accurate risk assessment and potentially lessen the sex disparities in treatment that currently exist.^3^^,^^10^ It was recently shown that both the GRACE 2.0 and GRACE 3.0 scores performed better in men than in women.^11^ On the other hand, a study including 831 women and 1,084 men showed that early discharge with a low HEART score appeared less safe for men than for women.^12^ A better understanding of sex differences in biomarkers can lead to greater understanding of biological differences that could be clinical beneficial for both sexes.

In this study, we investigated sex differences in the long-term prognostic value of circulating biomarkers including hs-cTn, hs-cTnI, NT-proBNP, GDF-15, and CRP, in patients presenting with acute chest pain. Since patients with and without ACS differ in pathophysiology and prognosis, we prospectively chose to perform subgroup analyses in patients with and without ACS.

## Methods

### Study sample

The prospective observational WESTCOR study (clinical trials number: NCT02620202) enrolled patients aged ≥18 years admitted with suspected NSTE-ACS to Haukeland University Hospital in Bergen, Norway, from 2015 to 2020. Additional details of the study have been published previously ^6^^,^^13^ and are provided in the Supplemental Appendix. Patients with missing biomarker concentrations were excluded from the current analytical cohort. In total, 1,476 patients were followed up for a median of 1,547 (IQR: 873-1,842) days (Central Illustration). The study was approved by the Regional Ethics Committee (REC number 2014/1,365), and the study conformed to the Declaration of Helsinki.

### Biochemical analysis

Blood samples were drawn at the emergency department at presentation and after 1, 3, and 8 to 12 hours. Hs-cTn (Elecsys) was measured in fresh, unfrozen serum, using an assay from Roche Diagnostics, Rotkreuz, Switzerland. Hs-cTnI was measured in biobanked serum using an assay from Abbott Diagnostics. For the statistical analysis, peak concentrations of hs-cTn and hs-cTnI during the primary hospitalization were used. The 1-hour sample was not obtained in the first one-third of included patients (this was preplanned and in accordance with the protocol due to logistics reasons). Approximately 97% had a sample after 3 hours, 94% had a sample after 8 to 12 hours, and 87% had both. In contrast, NT-proBNP, GDF-15, and CRP were analyzed only from biobanked admission serum samples stored at −80°C using assays from Roche Diagnostics. Details on assay characteristics are provided in the Supplemental Appendix.

### Definition of NSTE-ACS

Patients with NSTE-ACS included patients with NSTEMI and unstable angina. The adjudication process has been previously described.^13^ In brief, two independent cardiologists determined the final diagnosis by reviewing all available clinical data, routine laboratory results (including hs-cTn levels at admission and at 3 and 8-12 hours after admission), electrocardiograms, and imaging findings, including cardiac computed tomographic angiography and conventional coronary angiography. Any disagreements were resolved by a third adjudicator. Non-ST-segment elevation MI was defined according to the third universal definition for MI,^14^ a definition that remain unchanged in the fourth definition^15^ that was published after planning and onset of this study. A 20% (if the baseline hs-cTn concentration was >14 ng/L) or 50% (if the baseline hs-cTn concentration was ≤14 ng/L) change in troponin concentration was regarded significant. Unstable angina pectoris was defined as angina at rest with prolonged duration (>20 min), crescendo angina, recent destabilization of stable angina, or post-MI angina, with stable serial troponin concentrations and evidence of obstructive coronary artery disease.

### Clinical outcomes

The primary composite endpoint included all-cause mortality, incident MI, or hospitalization for heart failure, while a secondary endpoint included CV death. Follow-up data on readmittance, diagnoses, and procedures were collected through the Norwegian Patient Register while information on mortality was derived from the Norwegian Cause of Death Registry. The low excess mortality in Norway during the COVID-19 pandemic (before 2022), combined with the lack of significant differences in mortality rates before and after its onset in our dataset, negated the need for additional analyses on death rates during the pandemic.

### Statistical analysis

Categorical variables are presented as absolute numbers (%), while continuous variables are presented as median (IQR). Differences between groups were tested using the Pearson chi-square test or Fisher's exact test for categorical variables and Mann-Whitney U test for continuous variables. Linearity was examined using the Shapiro-Wilk test and q-q plots. Right skewed variables were log2-transformed to achieve normal distribution before being used in the secondary analysis including the Cox proportional hazard regressions models. Baseline biomarker concentrations are depicted in box-plots stratified by sex.

Cox proportional hazard regressions models were created to estimate unadjusted and adjusted HRs with 95% CIs for the association between different biomarker concentrations (log2 transformed) and study endpoints. The proportional hazard assumption was evaluated by plotting the residuals of covariates and the ranking of the failure times, and with *P* values >0.05 suggesting fulfilled criteria of assumptions. We created multiple Cox proportional hazards regressions models; model 1 was adjusted for age and estimated glomerular filtration rate (eGFR), while model 2 was adjusted for age, eGFR, hypertension, hyperlipidemia, diabetes, current smoking, and previous MI. The interaction between sex and study biomarkers was examined by entering a multiplicative term between sex and the investigated biomarker into the Cox proportional hazards regression models. The interaction analyses included both sex and the biomarker concentrations as covariates in addition to the interaction term. Biomarkers were investigated as continuous and categorical (>cutoff values) variables separately. For hs-cTn and hs-cTnI, the sex-neutral 99th percentile as specified by the manufacturer was used, in addition to the proposed sex-specific 99th percentiles (hs-cTn_peak_ >9 ng/L in women and >16 ng/L in men, hs-cTnI_peak_ >16 ng/L in women and >34 ng/L in men). For GDF-15, a cutoff of 1,200 pg/mL, which has been associated with increased risk in numerous studies,^6^^,^^7^^,^^16^ was used. For CRP, a cutoff of >2 mg/dL was chosen based on the American College of Cardiology (ACC)/American Heart Association (AHA) guidelines.^17^ Since there is no established prognostic cutoff for NT-proBNP, the 300-ng/L limit commonly employed to rule out acute heart failure was used. We fitted a generalized-additive-model smoothed spline using a Cox model to explore the association between biomarker concentrations and HRs and to visually display differences between men and women.

Discrimination was assessed by constructing receiver operating characteristic curves with corresponding area under the curve (AUC) for all biomarkers for their ability to predict the composite and secondary endpoints and compared using DeLong test. Optimal cutoff values for optimizing sensitivity and specificity for the prediction of primary endpoint were determined according to the Youden Index.

Subgroup analyses were performed for groups of patients with and without NSTE-ACS.

R (version.4.1.2), MedCalc statistical software version 17.6, and IBM SPSS Statistics for Windows (version.26.0) were used for statistical analysis. For all statistical testing, 2-sided *P* values were reported.

## Results

### Baseline characteristics

The study included 39.6% women (Table 1). Women were older than men (median age 65.0 vs 60.0 years), were more likely to be hypertensive, and had a higher prevalence of renal dysfunction. Men had more frequently hyperlipidemia, more often suffered a previous MI, had lower ejection fraction, and exhibited a higher prevalence of ACS during the index hospitalization. Prior to the index hospitalization, men were more commonly prescribed CV medications, including statins, beta-blockers, aspirin, and oral anticoagulants (Table 1).

**Table 1:** Baseline Characteristics of 1,476 Patients Admitted With Acute Chest Pain Stratified by Sex

	All Patients (N = 1,476)	Women (n = 584, 39.6%)	Men (n = 892, 60.4%)	*P* Value
Age, (y)	62 (51-72)	65 (54-76)	60 (50-70)	<0.001
Cardiovascular risk factors				
Obesity^a^	191 (26.4)	59 (21.9)	132 (29.1)	0.033
Active smoker	282 (19.1)	116 (19.9)	166 (18.6)	0.549
Hyperlipidemia^b^	571 (38.7)	190 (32.5)	381 (42.7)	<0.001
Diabetes mellitus	174 (11.8)	58 (9.9)	116 (13.0)	0.073
Hypertension^c^	605 (41.0)	263 (45.0)	342 (38.3)	0.011
Medical history				
Previous MI	283 (19.2)	75 (12.8)	208 (23.3)	<0.001
Atrial fibrillation	160 (10.8)	47 (8.0)	113 (12.6)	<0.001
Previous stroke	41 (2.8)	12 (2.1)	29 (3.3)	0.142
Family history of CAD	268 (18.2)	114 (19.5)	154 (17.3)	0.272
Renal failure^d^	183 (12.4)	92 (15.8)	91 (10.2)	0.002
Peripheral arterial disease	29 (2.0)	9 (1.5)	20 (2.2)	0.343
Known heart failure	51 (3.5)	18 (3.1)	33 (3.7)	0.525
Ejection fraction (N = 559)	58 (52-62)	60 (55-65)	56 (50-61)	<0.001
Laboratory parameters during index hospitalization				
Total cholesterol (mmol/L)	4.8 (3.9-5.8)	5.1 (4.3-6.2)	4.6 (3.7-5.6)	<0.001
LDL cholesterol (mmol/L)	3.1 (2.2-4.0)	3.2 (2.1-3.9)	3.0 (2.1-3.9)	<0.001
HDL cholesterol (mmol/L)	1.3 (1.1-1.7)	1.6 (1.3-2.0)	1.2 (1.0-1.5)	<0.001
eGFR (mL/min/1.73 m^2^)	86.0 (71.8-97.1)	88.0 (73.6-98.7)	83.2 (69.3-94.0)	<0.001
Diagnosis during initial admission				
NSTEMI	173 (11.7)	52 (8.9)	121 (13.6)	0.006
Unstable angina pectoris	196 (13.3)	57 (9.8)	139 (15.6)	0.001
ACS during index hospitalization	369 (25.0)	109 (18.7)	260 (29.1)	<0.002
Stable angina pectoris	14 (0.9)	9 (1.5)	5 (0.6)	0.057
Noncoronary cardiac disease	94 (6.4)	29 (5.0)	65 (7.3)	0.074
Noncardiac chest pain	898 (60.8)	390 (66.8)	508 (57.0)	<0.001
Medication at admission				
Aspirin	486 (32.9)	160 (27.4)	326 (36.5)	<0.001
Other antiplatelet	111 (7.5)	39 (6.7)	72 (8.1)	0.321
Oral anticoagulants	159 (10.8)	47 (8.0)	112 (12.6)	0.006
Warfarin	74 (5.0)	19 (3.3)	55 (6.2)	0.012
Beta-blocker	452 (30.6)	160 (27.4)	292 (32.7)	0.030
ACE inhibitor	495 (33.5)	192 (32.9)	303 (34.0)	0.664
Diuretic	260 (17.6)	110 (18.8)	150 (16.8)	0.319
Statin	562 (38.1)	184 (31.5)	378 (42.4)	<0.001

Values are median (IQR) or n (%).

IQR = interquartile range; MI = myocardial infarction; CAD = coronary artery disease; LDL = low-density lipoprotein; HDL = high-density lipoprotein; eGFR = estimated glomerular filtration rate; ACS = acute coronary syndrome; NSTEMI = non-ST-elevation myocardial infarction.

a Body mass index (BMI) >30 kg/m^2^, n = 394 are included for this variable.

b Use of lipid-lowering agents prior to admission.

c Use of antihypertensive medication at admission.

d Estimated glomerulation filtration rate (eGFR) <60 mL/min/1.73 m^2^.

### Sex differences in biomarker concentrations during hospitalization

**Figure 1:**
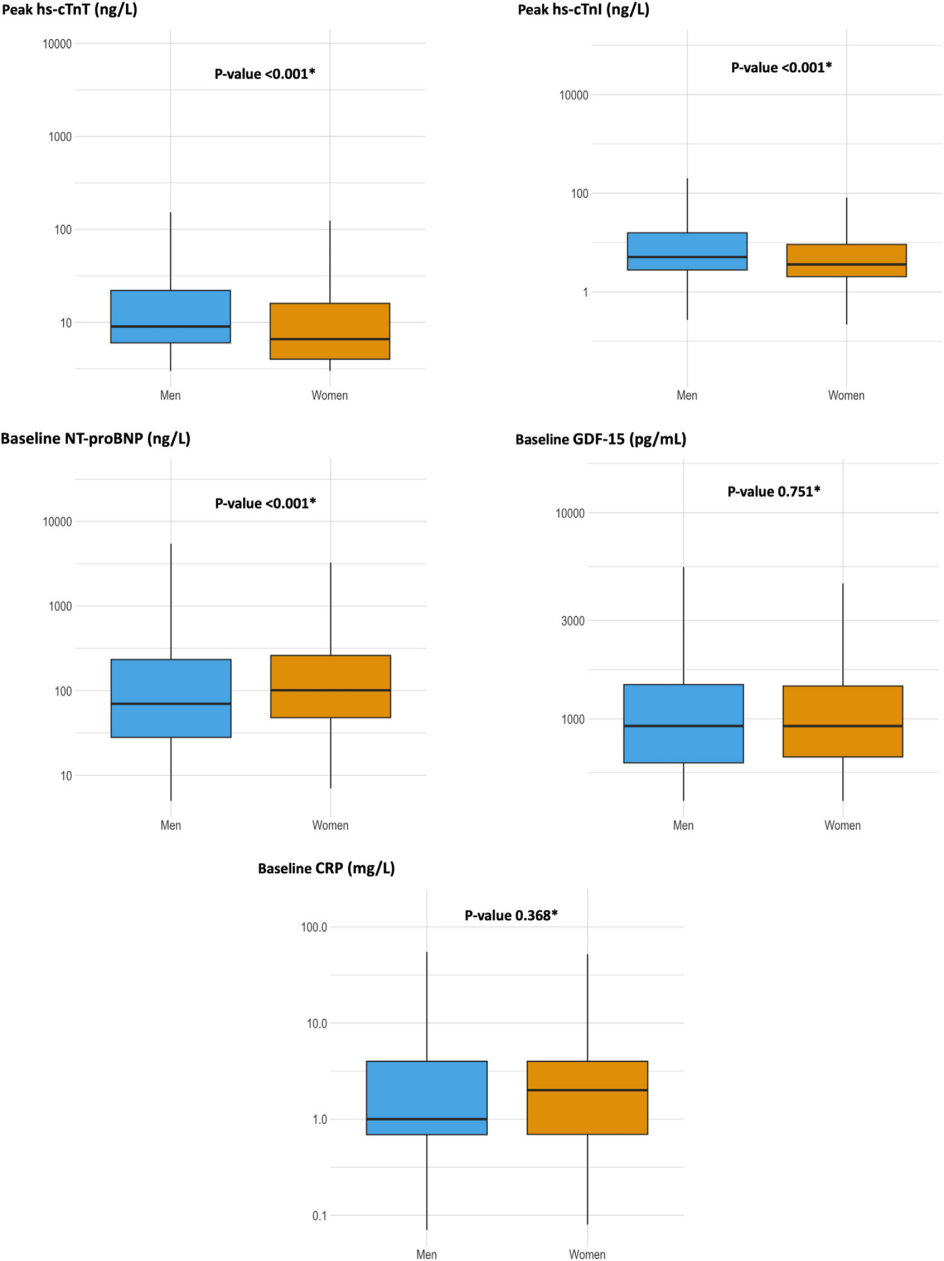
**Baseline Biomarker Concentrations Stratified by Sex** ∗*P* value for comparison of median concentrations using the Mann-Whitney U test.

Generally, hs-cTn and hs-cTnI concentrations were higher in men than in women, while NT-proBNP concentrations were higher in women than in men (Figure 1). We found no differences in GDF-15 and CRP concentrations between sexes (Figure 1).

### Clinical outcomes

During a median follow-up of 1,547 (IQR: 873-1,842) days, 14% of women and 14% of men reached the primary composite endpoint. The secondary endpoint occurred in 3% of women and 2% of men during a median follow-up of 1,660 (IQR: 1,219-1,965) days.

**Figure 2:**
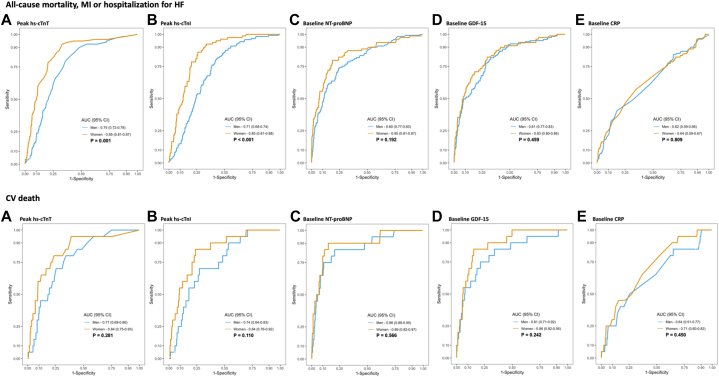
**Receiver Operating Characteristics Area Under the Curve Plotted for the Prediction of the Primary Endpoint: All-Cause Mortality, MI, or Hospitalization for HF and the Secondary Endpoint of Cardiovascular Mortality** Respective graphs for men (blue) and women (orange).

Biomarker concentrations had a high degree of discriminatory performance for the composite endpoint (Figure 2). Although the AUCs were numerically higher for all biomarkers in women than in men, the difference was only significant for hs-cTn (AUC: 0.85, 95% CI: 0.81-0.87 in women vs AUC: 0.75, 95% CI: 0.72-0.78 in men) and hs-cTnI (AUC: 0.85, 95% CI: 0.81-0.88 in women vs AUC: 0.71, 95% CI: 0.68-0.74 in men) (Figure 2). For both sexes, all biomarker concentrations except CRP had a high performance in discriminating CV death during total follow-up (Figure 2). Furthermore, all biomarker concentrations were associated with a numerically higher AUC in women than in men, but these differences were nonsignificant for the secondary endpoint (Figure 2).

### Composite endpoint

Increasing concentrations of all biomarkers were associated with an increased risk of the composite endpoint in both sexes although the estimates were numerically higher in women (Table 2, Central Illustration). In adjusted analyses, women had a higher risk of the composite endpoint than men for hs-cTn (HR: 1.3, 95% CI: 1.2-1.5 vs HR: 1.1, 95% CI: 1.0-1.2, *P* value for interaction = 0.009), hs-cTnI (HR: 1.2, 95% CI: 1.1-1.2 vs HR: 1.0, 95% CI: 1.0-1.1, *P* value for interaction = 0.005), and NT-proBNP (HR: 1.5, 95% CI: 1.3-1.7 vs HR: 1.3, 95% CI: 1.2-1.5, *P* value for interaction = 0.043). There was no difference in adjusted risk of the primary outcome with increasing concentrations of GDF-15 or CRP (Table 2). Optimal cutoff values for the prediction of the primary endpoint differed numerically between sexes for all biomarkers (Supplemental Table 1), and the largest relative difference was seen for NT-proBNP, being higher in women (239 pg/L) than in men (171 pg/L).

**Table 2:** HR (95% CI) in Proportional Hazards Regressions for the Associations of log2-Transformed Biomarkers in Relation to the Composite Endpoint, and Secondary Endpoint in Unadjusted and Adjusted (Current Smoking, Age, Diabetes, Hypertension, Hyperlipidemia, eGFR, and Previous MI) Models

HR (95% CI) for Primary Endpoint of All-Cause Mortality, MI, or Hospitalization for HF						
	Women	Men	*P* Value (Interaction)^a^	Women	Men	*P* Value (Interaction)^a^
	Unadjusted			Adjusted		
Log2 hs-cTn_peak_	1.4 (1.3-1.5) *P* < 0.001	1.2 (1.2-1.3) *P* < 0.001	0.003	1.3 (1.2-1.5) *P* < 0.001	1.1 (1.0-1.2) *P* = 0.016	0.009
Log2 hs-cTnI_peak_	1.2 (1.2-1.3) *P* < 0.001	1.1 (1.1-1.2) *P* < 0.001	0.001	1.2 (1.1-1.2) *P* < 0.001	1.0 (1.0-1.1) *P* = 0.332	0.005
Log2 NT-proBNP_BL_	1.8 (1.6-1.9) *P* < 0.001	1.5 (1.4-1.6) *P* < 0.001	0.008	1.5 (1.3-1.7) *P* < 0.001	1.3 (1.2-1.5) *P* < 0.001	0.043
Log2 GDF-15_BL_	2.9 (2.4-3.5) *P* < 0.001	2.9 (2.4-3.4) *P* < 0.001	0.842	2.1 (1.6-2.8) *P* < 0.001	2.1 (1.6-2.6) *P* < 0.001	0.404
Log2 CRP_BL_	1.3 (1.2-1.4) *P* < 0.001	1.2 (1.1-1.3) *P* < 0.001	0.540	1.2 (1.1-1.3) *P* = 0.003	1.2 (1.1-1.3) *P* < 0.001	0.622

**HR (95% CI) for Secondary Endpoint of CV Death**						
	Unadjusted			Adjusted^b^		
Log2 hs-cTn_peak_	1.5 (1.3-1.7) *P* < 0.001	1.3 (1.1-1.5) *P* < 0.001	0.215	1.3 (1.1-1.6) *P* < 0.001	1.2 (1.0-1.4) *P* = 0.101	0.384
Log2 hs-cTnI_peak_	1.3 (1.2-1.4) *P* < 0.001	1.2 (1.1-1.3) *P* = 0.004	0.170	1.2 (1.1-1.3) *P* = 0.004	1.1 (1.0-1.2) *P* = 0.204	0.354
Log2 NT-proBNP_BL_	1.9 (1.6-2.2) *P* < 0.001	1.7 (1.4-2.0) *P* < 0.001	0.320	1.7 (1.4-2.2) *P* < 0.001	1.5 (1.2-1.9) *P* < 0.001	0.480
Log2 GDF-15_BL_	3.1 (2.2-4.2) *P* < 0.001	3.0 (2.1-4.4) *P* < 0.001	0.969	2.7 (1.7-4.3) *P* < 0.001	2.3 (1.4-3.8) *P* = 0.002	0.806
Log2 CRP_BL_	1.4 (1.2-1.7) *P* < 0.001	1.3 (1.0-1.6) *P* < 0.001	0.492	1.3 (1.1-1.6) *P* = 0.014	1.2 (0.9-1.4) *P* = 0.183	0.719

CRP = C-reactive protein; CV = cardiovascular; GDF-15 = growth differentiation factor-15; HF = heart failure; hs-cTn = high-sensitivity cardiac troponin; hs-cTnI = high-sensitivity cardiac troponin I; MI = myocardial infarction; NT-proBNP = N-terminal pro-B-type natriuretic peptide.

a Interaction term between sex and biomarker (log2-tranformed) were entered into the cox regression model as a multiplicative term.

b Adjusted for age and estimated glomerular filtration rate (eGFR).

**Central Illustration:**
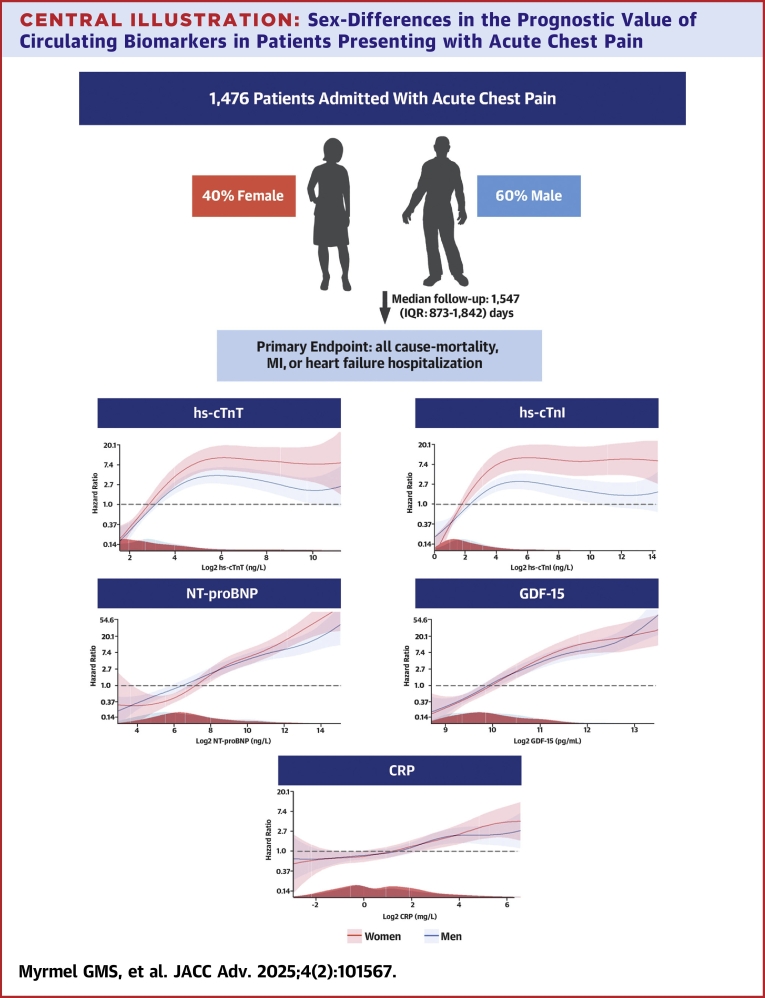
**Sex-Differences in the Prognostic Value of Circulating Biomarkers in Patients Presenting with Acute Chest Pain** Study flow chart and generalized additive models (GAM) curves demonstrating unadjusted HRs with 95% CIs (red = women; blue = men) for different endpoints along the y-axis and log2-transformed biomarker concentrations along the x-axis. Density plots demonstrating the distribution of biomarker concentrations (red = women; blue = men) are shown on the x-axis.

### CV death

All circulating biomarkers were associated with an increased risk of CV death in both men and women. However, we found no interaction between sex and any of the biomarkers with regards to predicting CV death (Table 2).

### Use of prespecified cutoffs

Using the sex-neutral 99th percentile cutoff for myocardial injury for the hs-cTn assay, we found a significant difference in risk between women and men, with women carrying the highest risk (HR: 3.1, 95% CI: 1.8-5.6 in women vs HR: 1.8, 95% CI: 1.1-2.8 in men) (Table 3). A similar difference was observed for the sex-neutral 99th percentile cutoff for the hs-cTnI assay (HR: 2.2, 95% CI: 1.4-3.7 in women vs HR: 1.1, 95% CI: 0.8-1.7 in men). The 99th percentile sex specific cutoff values for hs-cTn and hs-cTnI were also associated with higher risk in women than in men (Table 3), and this was particularly clear for hs-cTn. Using the 99th percentile sex-specific cutoff values for hs-cTn and hs-cTnI, we found a significant difference in AUC, with better discrimination for the primary endpoint in women than in men (Supplemental Table 2). There was no significant interaction between sex and the NT-proBNP cutoff 300 ng/L, GDF-15 cutoff 1,200 pg/mL, or CRP cutoff 2 mg/L.

**Table 3:** Cox Proportional Hazard Regressions Models for the Association of Prespecified Cutoffs for Biomarkers and the Primary Outcome

HR (95% CI) for All-Cause Mortality, MI, and Hospitalization for HF						
	Unadjusted			Adjusted^a^		
	Women	Men	*P* Value (Interaction)	Women	Men	*P* Value (Interaction)
hs-cTn_peak_ >14 ng/L	8.5 (5.2-13.9) *P* < 0.001	4.6 (3.1-6.7) *P* < 0.001	<0.001	3.1 (1.8-5.6) *P* < 0.001	1.8 (1.1-2.8) *P* = 0.014	0.045
hs-cTn_peak_ (sex-specific cutoffs^b^)	20.7 (9.0-47.7) *P* < 0.001	4.4 (3.0-6.1) *P* < 0.001	<0.001	8.7 (3.5-21.6) *P* < 0.001	1.8 (1.2-2.8) *P* < 0.007	0.003
hs-cTnI_peak_ >28 ng/L	5.4 (3.4-8.2) *P* < 0.001	2.1 (1.4-3.0) *P* < 0.001	0.002	2.2 (1.4-3.7) *P* = 0.001	1.1 (0.8-1.7) *P* = 0.523	0.017
hs-cTnI_peak_ (sex-specific cutoffs^c^)	5.3 (3.4-8.2) *P* < 0.001	2.0 (1.4-3.0) *P* < 0.001	<0.001	2.3 (1.4-3.8) *P* < 0.001	1.2 (0.8-1.8) *P* = 0.408	0.018
NT-proBNP_BL_ >300 ng/L	9.9 (6.0-16.1) *P* < 0.001	6.8 (4.7-9.7) *P* < 0.001	0.454	3.5 (1.9-6.2) *P* < 0.001	3.1 (2.1-4.7) *P* < 0.001	0.365
GDF-15_BL_ >1,200 pg/mL	7.4 (4.4-12.3) *P* < 0.001	7.2 (4.7-11.1) *P* < 0.001	0.968	2.2 (1.2-4.2) *P* = 0.014	2.4 (1.5-4.1) *P* = 0.001	0.743
CRP_BL_ >2 mg/L	2.3 (1.5-3.6) *P* < 0.001	1.9 (1.4-2.8) *P* < 0.001	0.548	1.9 (1.2-3.0) *P* = 0.007	1.5 (1.0-2.2) *P* = 0.032	0.383

BL = baseline.

a Adjusted for current smoking, age, diabetes, hypertension, hyperlipidemia, estimated glomerular filtration rate, and previous MI.

b hs-cTn_peak_ >9 ng/L in women, >16 ng/L in men.

c hs-cTnI_peak_ >16 ng/L in women, >34 ng/L in men.

### Subgroup analysis comparing the risk in patients with and without NSTE-ACS

In patients with NSTE-ACS during the index hospitalization, 27% of women (n = 109) and 23% of men (n = 206) met the composite endpoint. In patients without NSTE-ACS during the index hospitalization, 12% of men (n = 632) and 11% of women (n = 475) met the composite endpoint. Biomarker concentrations in patients with and without NSTE-ACS stratified by sex are shown in Supplemental Table 3. In patients with NSTE-ACS, overall HRs associated with a doubling of all biomarker concentrations were numerically higher in women than in men, but the interaction by sex was only significant for hs-cTnI (Table 4). In patients without NSTE-ACS, the impact of sex on the prognostic ability of NT-proBNP was significant, with the risk being higher in women. This significant difference prevailed after adjusting for age and eGFR (Table 4). For hs-cTn and hs-cTnI, the AUC was lower in men than in women in both patient subgroups, those with NSTE-ACS and those without NSTE-ACS (Table 5). For NT-proBNP, GDF-15, and CRP, we observed no significant differences in AUCs between patients with NSTE-ACS and those without NSTE-ACS (Table 5).

**Table 4:** HR (95% CI) in Proportional Hazards Analyses Were Calculated to Evaluate the Associations Between log2-Transformed Biomarker Concentrations and the Primary Composite Endpoint, Both in Unadjusted Models and a Model Adjusted for Age and eGFR, Stratified by Patients With and Without NSTE-ACS

HR (95% CI) for Primary Endpoint of All-Cause Mortality, MI, or Hospitalization for HF						
	With NSTE-ACS (n = 369)			Without NSTE-ACS (n = 1,107)		
	Women (n = 109)	Men (n = 260)	*P* Value^b^ (Interaction)	Women (n = 475)	Men (n = 632)	*P* Value^b^ (Interaction)
Unadjusted						
Log2 hs-cTn_peak_	1.2 (1.1-1.4) *P* = 0.002	1.1 (1.0-1.2) *P* = 0.062	0.160	1.6 (1.5-1.8) *P* < 0.001	1.6 (1.4-1.7) *P* < 0.001	0.524
Log2 hs-cTnI_peak_	1.1 (1.1-1.2) *P* = 0.002	1.03 (1.0-1.1) *P* = 0.424	0.065	1.3 (1.2-1.4) *P* < 0.001	1.3 (1.2-1.4) *P* < 0.001	0.406
Log2 NT-proBNP_BL_	1.5 (1.2-1.7) *P* < 0.001	1.4 (1.3-1.6) *P* < 0.001	0.778	1.9 (1.7-2.2) *P* < 0.001	1.5 (1.4-1.7) *P* < 0.001	0.004
Log2 GDF-15_BL_	2.4 (1.5-3.8) *P* < 0.001	2.9 (2.1-3.9) *P* < 0.001	0.546	3.2 (2.6-4.0) *P* < 0.001	2.8 (2.3-3.4) *P* < 0.001	0.363
Log2 CRP_BL_	1.2 (1.0-1.4) *P* = 0.097	1.2 (1.0-1.4) *P* = 0.024	0.830	1.3 (1.2-1.5) *P* < 0.001	1.2 (1.1-1.4) *P* < 0.001	0.440
Adjusted^a^						
Log2 hs-cTn_peak_	1.3 (1.1-1.5) *P* = 0.002	1.1 (0.9-1.2) *P* = 0.318	0.073	1.4 (1.2-1.6) *P* < 0.001	1.4 (1.2-1.7) *P* < 0.001	0.751
Log2 hs-cTnI_peak_	1.2 (1.1-1.3) *P* < 0.001	1.0 (0.9-1.1) *P* = 0.789	0.024	1.2 (1-1.3) *P* = 0.018	1.2 (1-1.3) *P* = 0.023	0.682
Log2 NT-proBNP_BL_	1.4 (1.2-1.7) *P* < 0.001	1.2 (1-1.4) *P* = 0.021	0.263	1.6 (1.3-1.9) *P* < 0.001	1.4 (1.3-1.5) *P* < 0.001	0.029
Log2 GDF-15_BL_	1.8 (0.9-3.2) *P* = 0.077	2.2 (1.5-3.2) *P* < 0.001	0.451	2.5 (1.9-3.4) *P* < 0.001	2.1 (1.6-2.8) *P* < 0.001	0.101
Log2 CRP_BL_	1.2 (1.0-1.4) *P* = 0.052	1.1 (1.0-1.3) *P* = 0.020	0.431	1.2 (1.1-1.4) *P* = 0.004	1.2 (1.1-1.3) *P* = 0.003	0.421

BL = baseline; NSTE-ACS = non-ST-segment elevation-acute coronary syndrome.

a Adjusted for age and estimated glomerular filtration rate.

b Interaction term between sex and the study biomarker entered into the Cox proportional regressions model as a multiplicative term.

**Table 5:** Receiver Operating Characteristics-Area Under the Curve Estimates for Biomarkers Predicting and Discriminating the Primary Outcome

Area Under the Curve (95% CI) for All-Cause Mortality, MI and Hospitalization for HF						
	NSTE-ACS (n = 369)			Without NSTE-ACS (n = 1,107)		
	Women (n = 109)	Men (n = 260)	AUC Difference	Women (n = 475)	Men (n = 632)	AUC Difference
hs-cTn_peak_	0.74 (0.64-0.84)	0.60 (0.52-0.68)	0.13 (0.00-0.26) *P* = 0.044	0.87 (0.81-0.92)	0.81 (0.76-0.86)	0.05 (−0.02 to 0.13) *P* = 0.054
hs-cTnI_peak_	0.75 (0.66-0.84)	0.56 (0.48-0.65)	0.19 (0.06-0.31) *P* = 0.004	0.85 (0.81-0.90)	0.78 (0.74-0.82)	0.07 (0.01-0.14) *P* = 0.035
NT-proBNP_BL_	0.79 (0.70-0.89)	0.71 (0.63-0.80)	0.08 (−0.05 to 0.21) *P* = 0.231	0.85 (0.78-0.91)	0.83 (0.78-0.88)	0.02 (−0.07 to 0.10) *P* = 0.730
GDF-15_BL_	0.70 (0.58-0.82)	0.79 (0.71-0.86)	0.09 (−0.05 to 0.23) *P* = 0.211	0.88 (0.83-0.93)	0.81 (0.76-0.86)	0.07 (0.0-0.14) *P* = 0.050
CRP_BL_	0.61 (0.49-0.73)	0.59 (0.49-0.69)	0.02 (−0.14 to 0.18) *P* = 0.787	0.65 (0.56-0.73)	0.64 (0.58-0.71)	0.01 (−0.11 to 0.11) *P* = 0.963

Differences in AUCs are provided between patients with non-ST-segment elevation-acute coronary syndrome (NSTE-ACS) and those without NSTE-ACS.

BL = baseline.

## Discussion

In this study, we demonstrate that increasing concentrations of cTn and NT-proBNP are associated with a higher risk of death and CV events in women than in men, even after adjusting for age and traditional CV risk factors. These cardiac biomarkers also carried a higher discriminative power in women than in men. Increasing concentrations of GDF-15 and CRP were associated with similar risk in men and women. Women with hs-cTn and hs-cTnI above the sex-neutral cutoff carried a higher risk for CV events after adjusting for traditional clinical factors, a difference that was even larger when sex-specific cutoffs were used (hs-cTn). Our findings may have significant implications in long-term risk stratification and for the interpretation of cardiac risk calculators and predictors when employing hs-cTn and NT-proBNP in patients presenting with acute chest pain given the notable sex differences. Failure to account for such differences in risk can potentially contribute to some of the undertreatment of CV risk that has earlier been observed in women.^3^^,^^4^^,^^10^

### Impact of sex on the prognostic value of cTn

Several studies have shown that cTn are stronger predictors of CV events in women than in men in the general population.^8^^,^^9^ However, the literature on the impact of sex on the long-term prognosis in patients presenting with acute chest pain is limited. In line with our results, a study by Eggers et al^18^ including 2,750 patients with NSTE-ACS showed that cTnI exhibited stronger prognostic information in women than in men when investigating the risk of 30-day MI and death and 1-year risk of death. Conversely, a recent register-based study including 48,250 patients with suspected ACS could not show that the use of sex-specific cTn cutoff concentrations changed the 1-year risk prediction.^19^ In our study, absolute concentrations of hs-cTn and hs-cTnI were associated with a higher risk of death and CV events in women than in men, and there was also a sex difference in discriminative power as demonstrated by the difference in AUC. The increased risk associated with hs-cTn elevations in women also persisted after adjusting for age and traditional risk factors, demonstrating that the increased risk seen in women is not merely a result of increased comorbidity and other CV risk factors. The sex-neutral and sex-specific 99th percentiles for hs-cTn and hs-cTnI were also associated with higher risk in women than in men, meaning that when women and men are measured at the same absolute concentration, women have higher long-term CV risk than men. Using the sex-specific cutoff for myocardial injury moves men further away and women closer to the optimal cutoff for long-term risk prediction. These findings also show that a lower cutoff value than the 99th percentile cTn for myocardial injury should be employed for long-term risk prediction, aligning with studies from the general population.^9^^,^^20^ There are several reasons why increasing cTn concentrations are associated with higher risk in women. cTn concentrations tend to be lower in women than in men at a given age, and a similar increase in cTn concentration is thus relatively higher in women than in men. The lower cTn concentrations seen in women is partly due to lower left ventricular mass,^21^, ^22^, ^23^ the cardioprotective effects of estrogen,^24^ and because men tend to develop hypertension earlier than women.^25^ Women reach similar concentrations of cTn as men nearly a decade later, but the relative increase with age is higher in women than in men. This lag in concentration is partially explained by sex variations in the aging process in the myocardium and the vasculature.^26^^,^^27^ Our data suggest that the well-known sex differences in cTn concentrations have prognostic implications that should be reflected in clinical practice.

### Impact of sex on the prognostic value of NT-proBNP

The 2020 European Society of Cardiology (ECS) guidelines on NSTEMI-ACS state that natriuretic peptides could be used to obtain prognostic information and is assigned a class IIa, level B recommendation.^28^ NT-proBNP concentrations are generally higher in women than in men,^29^ but the risk associated with increasing concentrations of NT-proBNP in patients with heart failure seems to be similar in men and women.^30^^,^^31^ A knowledge gap is still present on how sex impacts the prognostic value of NT-proBNP in patients presenting with suspected NSTE-ACS. In our study, baseline concentrations of NT-proBNP were higher in women, and a doubling of NT-proBNP was associated with a higher risk of the composite endpoint in women than in men in both unadjusted and adjusted analysis. The discriminative power assessed by AUC was estimated at 0.85 in women and 0.80 in men for the composite endpoint, but this difference was not significant. In addition, the optimal cutoff value for the prediction of the primary endpoint differed between sexes, being higher in women (239 pg/L) than in men (171 pg/L), which again may be attributed to the inherently higher NT-proBNP levels seen in healthy women. These findings indicate that increasing NT-proBNP concentrations may be associated with higher CV risk in women than in men among patients presenting with suspected NSTE-ACS. Although these findings were less clear than the association with cTn, this still seems warranted to consider when NT-proBNP is used for prognostication in this setting.

### Impact of sex on the prognostic value of GDF-15

Although not routinely used for risk stratification in patients presenting with suspected ACS, studies have shown that GDF-15 is a strong prognostic marker in this setting.^6^^,^^7^^,^^16^ Research on sex differences of GDF-15 in general is scarce, and to our knowledge, this is the first study to evaluate sex differences in the prognostic value of this marker in acute chest pain patients. GDF-15 concentrations were associated with increased risk of the composite endpoint in both sexes. There was no impact of sex on the prognostic value of GDF-15 in predicting death or CV events. These findings suggest that GDF-15 can be used as a prognostic marker in both men and women, and that the prognostic value is equal for both sexes and aligns with studies performed in community-dwelling individuals.^32^

### Impact of sex on the prognostic value of CRP

CRP has been linked to vascular inflammation and different CV disease manifestations such as MI for decades. Numerous studies have shown that CRP can be used for CV risk prediction, and in the 2019 ACC/AHA guideline on the primary prevention and CV disease, CRP >2 mg/L is listed as a risk-enhancing factor.^17^ Previous research on whether there are sex differences in the predictive ability of CRP on CV risk has been diverging,^33^, ^34^, ^35^ and the literature on acute chest pain patients is limited. In our study, CRP was an independent predictor of the composite endpoint in unadjusted and adjusted models in both men and women. The interaction between sex and CRP was nonsignificant in the prediction of the composite endpoint and CV death suggesting that CRP carries equal prognostic value in men and women. CRP >2 mg/L was associated with numerically higher adjusted HR (1.9, 95% CI: 1.2-3.0) in women than in men (HR: 1.5, 95% CI: 1.0-2.2), but the interaction by sex was nonsignificant. Our findings suggest that CRP can be used as a prognostic marker in acute chest pain patients regardless of sex and in patients with and without ACS during admission.

### Clinical implications

Women presenting with suspected ACS may be undertreated with regards to CV risk factors,^4^ and sex-specific cutoffs could be employed for several biomarkers, including cTn and NT-proBNP, when used for long-term prognostication in patients presenting with acute chest pain. Several investigators have advocated using cTn and other circulating biomarkers on a continuous scale rather than dichotomized for prognostication, and also here, the impact of sex needs to be considered for adequate risk stratification, including the commonly used risk calculators. GDF-15 and CRP seemingly can be used for risk stratification in patients with acute chest pain regardless of sex, and sex-specific cutoffs and sex-adjustment do not seem warranted. Further research is needed to determine which risk-assessment tools are best suited, and how it can translate into improved patient care, including who will benefit from risk-reduction therapies such as statins and lifestyle modifications, and also how the impact of sex on the prognostic ability of circulating biomarkers shall be used to ensure equality in risk assessment for both men and women.

### Strengths and limitations

This study has several strengths, including a high proportion of women (40%). Biomarkers reflecting different pathophysiological pathways were evaluated, including myocardial injury markers, cardiac hormones like natriuretic peptides, and inflammatory markers. Strong event capture was also ensured by collecting endpoints from a register including mandatory registration of all Norwegian health care contacts. The study also has some limitations. The subgroups, including patients with NSTE-ACS, were small, and this analysis might be underpowered. Due to fewer endpoints, it was only possible to adjust for model 1, which may exclude potential confounders. The low number of CV deaths may have reduced the statistical power to detect significant differences, and larger studies will be necessary to validate these findings. In addition, NT-proBNP and the inflammatory markers were measured at baseline only, and thus, it was not possible to investigate if the peak concentration of these biomarkers showed different properties. In addition, echocardiography data were available for only 559 patients, with ejection fraction being the only reported parameter.

## Conclusions

In patients presenting with acute chest pain cTn, troponin I and NT-proBNP are stronger predictors of long-term death and CV events in women than in men. Increasing concentrations of GDF-15 and CRP carry similar risk of death and CV events in men and women.

## Funding Support and Author Disclosures

This study was financed by a grant from the 10.13039/501100004257Western Norway Regional Health Authority (grant number: 912265). Dr G.M.S. Myrmel has had a part time research grant from 10.13039/100016190Trond Mohn Foundation and currently is receiving a Ph.D. grant from the 10.13039/501100004257Western Norway Regional Health Authority (grant number: F-12589). The reagent costs for GDF-15 were covered by Roche Diagnostics. The sponsor had no influence on the analyzing or interpretation of the data, nor on the writing of the manuscript. Dr Aakre has served on the advisory board of Roche Diagnostics, Siemens Healthineers, and SpinChip; consultant honoraria from CardiNor; lecturing honorarium from Siemens Healthineers, Roche Diagnostics, Mindray, and Snibe Diagnostics; and research grants from Siemens Healthineers and Roche Diagnostics. She is an Associate Editor of Clinical Biochemistry and Chair of the IFCC Committee of Clinical Application of Cardiac Bio-markers. Dr Omland has received speaker and/or consultancy honoraria from Abbott Diagnostics, Bayer, CardiNor, Roche Diagnostics, and Siemens Healthineers and has received research support from Abbott Diagnostics, Novartis, Roche Diagnostics, via Akershus University Hospital. Dr Skadberg has received lecture fees from Abbott Diagnostics. All other authors have reported that they have no relationships relevant to the contents of this paper to disclose.
