# Supplementary material for: Sex Differences in the Prognostic Value of Circulating Biomarkers in Patients Presenting With Acute Chest Pain
Source: JACC Adv. 2025 Jan 21;4(2):101567. doi: 10.1016/j.jacadv.2024.101567 (PMC11791240; doi:10.1016/j.jacadv.2024.101567)
Supplement: Supplemental Material [file mmc1.docx]

**Supplemental Appendix**

**Biochemical analysis:** The Roche Diagnostics hs-cTnT assay has a limit of blank (LoB) of 3 ng/L, a limit of detection (LoD) of 5 ng/L and an analytical variation (CV_A_) of 10% or lower for concentrations >4.5 ng/L. The 99th percentile URL is 9.0 ng/L in women and 16.8 ng/L in men. The hs-TnI measurements were done using the Architect and Alinity instruments from Abbott Diagnostics. Architect had a LoB at 0.7 ng/L and an LoD of 1.1 ng/L, whilst Alinity had a LoB of 1.0 ng/L and a LoB of 1.6 ng/L. Both had a 10% CV_A_ at 4.7 ng/L and 99th percentile URL of 15.6 ng/L in women and 34.2 ng/L in men. The GDF-15 assay (Roche Diagnostics) has a measuring range from 400 (LoD) to 20 000 pg/mL; the highest CV_A_ was 2% at concentration 520 pg/mL. The CRP assay was from Roche Diagnostics, with a measuring range of 1-350 mg/L and had a CV_A_ <5%.

**Supplemental Table 1:** Optimal cut-off values for prediction of the primary endpoint (All-cause mortality, incident myocardial infarction and heart failure hospitalization) were determined according to the Youden index in men and women. Also shown is the sensitivity and specificity for the cut-off values.

| **Youden Index and optimal cut-off values for optimizing sensitivity**  **and specificity for the prediction of the primary endpoint** | | | | |
| --- | --- | --- | --- | --- |
|  | **Men** | | **Women** | |
|  | Optimal cut-off value (sensitivity/specificity) | Youden  Index | Optimal cut-off value  (sensitivity/specificity) | Youden  Index |
| **hs-cTnT_peak_** | >11ng/L (79/64) | 0.44 | >8ng/L (92/67) | 0.59 |
| **hs-cTnI_peak_** | >5.4 ng/L (81/57) | 0.38 | >6.1ng/L (86/74) | 0.60 |
| **NT-proBNP_BL_** | >171 ng/L (74/76) | 0.49 | >239ng/l (80/81) | 0.61 |
| **GDF-15_BL_** | >1203 pg/mL (78/71) | 0.49 | >1433 pg/mL (71/82) | 0.53 |
| **CRP_BL_** | >4 mg/L (41/82) | 0.23 | >2 mg/L (57/65) | 0.22 |

**Supplemental Table 2:** Receiver operating characteristics-area under the curve (ROC-AUC) estimates for biomarker cut-off values predicting and discriminating the primary outcome. Differences in AUCs are provided between men and women.

| **Area under the curve (95% confidence interval) for all-cause mortality, MI and hospitalization for HF** | | | | |
| --- | --- | --- | --- | --- |
|  | All | Men | Women | Difference in C-statistic |
| **hs-cTnT_peak_ >14ng/L** | 0.72 (0.68 – 76) | 0.70 (0.64 – 0.75) | 0.76 (0.70 – 0.82) | 0.07 (-0.01 – 0.15), p = 0.094 |
| **hs-cTnT_peak_**  **(**Sex specific cut-offs†) | 0.73 (0.70 – 0.77) | 0.70 (0.64 – 0.75) | 0.80 (0.75 – 0.84) | 0.10 (0.04 – 0.17), p = 0.003 |
| **hs-cTnI_peak_ >28 ng/L** | 0.61 (0.56 – 0.65) | 0.57 (0.52 – 0.63) | 0.66 (0.59 – 0.73) | 0.09 (-0.01 – 0.18), p = 0.069 |
| **hs-cTnI_peak_**  **(**Sex specific cut-offs#) | 0.61 (0.57 – 0.66) | 0.57 (0.51 – 0.63) | 0.68 (0.61 – 0.75) | 0.11 (0.02 – 0.20), p = 0.019 |

† hs-cTnT_peak_ >9ng/L in women, >16ng/L in men

#hs-cTnI_peak_ >16 ng/L in women, >34 ng/L in men.

**Supplemental Table 3:** Table showing biomarker concentrations in men and women with and without NSTE-ACS during index hospitalization. In patients with NSTE-ACS, NT-proBNP concentrations were higher in women compared to men. In patients without NSTE-ACS peak hs-TnT and peak hs-TnI concentrations were higher in men compared to women, while NT-proBNP concentrations were higher in women.

| **Biomarker concentrations (median, IQR) in patients without NSTE-ACS (n = 1107)** | | | | |
| --- | --- | --- | --- | --- |
|  | **Total** | **Women** | **Men** | **p-value** |
| **hs-cTnT_peak_ ng/L** | 7 (4 – 12) | 6 (3 - 11) | 7 (5 – 13) | <0.001 |
| **hs-cTnI_peak_ ng/L** | 4 (2 -7) | 3 (2 – 7) | 4 (3-8) | <0.001 |
| **NT-proBNP_BL_ ng/L** | 70 (30 – 182) | 89 (46 – 199) | 53 (22 – 168) | <0.001 |
| **GDF-15_BL_ pg/mL** | 861 (596 – 1332) | 879 (629 – 1370) | 843 (572 – 1318) | 0.172 |
| **CRP_BL_ mg/L** | 1 (1-4) | 2 (1- 4) | 1 (1 – 4) | 0.257 |
| **Biomarker concentrations (median, IQR) in patients with NSTE-ACS (n = 369)** | | | | |
| **hs-cTnT_peak_ ng/L** | 28 (9 – 192) | 26 (7 – 482) | 28 (11 - 214) | 0.169 |
| **hs-cTnI_peak_ ng/L** | 28 (5 – 723) | 19 (4 – 611) | 35 (5-811) | 0.133 |
| **NT-proBNP_BL_ ng/L** | 167 (60 -595) | 237 (89 – 760) | 152 (57 – 516) | 0.026 |
| **GDF-15_BL_ pg/mL** | 1148 (756 - 1719) | 1034 (784 – 1949) | 1182 (747 – 1647) | 0.934 |
| **CRP_BL_ mg/L** | 2 (1 – 4) | 2 (1 – 5) | 2(1 – 4) | 0.785 |
